# *Mycobacterium celatum* Pulmonary Infection in the Immunocompetent: Case Report and Review

**DOI:** 10.3201/eid0903.020342

**Published:** 2003-03

**Authors:** Claudio Piersimoni, Pier Giorgio Zitti, Domenico Nista, Stefano Bornigia

**Affiliations:** * Umberto I°–Torrette Hospital, Ancona, Italy

**Keywords:** *Mycobacterium celatum*, pulmonary infection, immunocompetent host, dispatch

## Abstract

*Mycobacterium celatum* has been shown to cause disease in immunocompromised patients. We report a case of serious pulmonary infection caused by *M. celatum* in an apparently immunocompetent patient and review the characteristics of two other reported cases. Clinical and radiologic symptoms and signs included cough, malaise, and weight loss associated with cavitary lesions and pulmonary infiltrates. Although *M. celatum* is easy to detect in clinical specimens by liquid and solid media, it may be misidentified as a member of the *M. tuberculosis* complex or as *M. xenopi*. *M. celatum* pulmonary infection appears to respond to antimycobacterial chemotherapy, particularly with clarithromycin.

*Mycobacterium celatum* was first described in 1993 as a new species whose mycolic acid pattern closely resembled that of *M. xenopi* (xenopi-like) but was biochemically indistinguishable from *M. avium* complex (MAC) (*1*). By sequencing studies of the 16S rRNA gene, now known to exist in two different copies within *M. celatum* genome (*2*), three different types could be differentiated (*1*,*3*). Cross-reactivity with the Accuprobe assay (Gen-Probe Inc., San Diego, CA) for the *M. tuberculosis* complex (MTB) was observed by probing types 1 and 3 but not type 2 (*3*,*4*). DNA sequencing showed that *M. celatum* type 1 differs by one nucleotide from the probe used in the Accuprobe assay, type 2 differs by four nucleotides (*5*), and the type 3 DNA sequence for the probe region is unknown.

Although *M. celatum* has been reported to cause infection mainly in AIDS patients (*6*–*10*), recently a growing amount of clinical evidence indicates that the infection can lead to serious disease in immunocompetent subjects as well (*11*–*13*). In this case, the principles for diagnosis of diseases caused by nontuberculous mycobacteria, recently updated by the American Thoracic Society (*14*), need to be properly followed. We report a well-documented case of a pulmonary disease with this mycobacterium in an apparently immunocompetent woman and review the characteristics of two similar published cases.

## The Study

A 63-year-old, HIV-negative woman was referred in June 2000 by a general practitioner to the outpatient pulmonary service because she had exhibited a fever and night sweats for 3 months. The patient’s medical history included a mild hypertensive cardiovascular disease and a pulmonary tuberculosis episode when she was 16 years old. Her father and brother had pulmonary tuberculosis. She lived in an urban area and had no history of acoholism, smoking, or use of steroids or immunosuppressive drugs. No other pulmonary diseases that could potentially lead to bronchiectasis and further mycobacterial colonization were reported***.*** Physical examination revealed no pathologic findings except a strong positive reaction (24 mm of induration) with tuberculin skin test (5 U of purified protein derivative–standard [PPD-S]). Results of clinical laboratory tests were unremarkable, apart from an elevated erythrocyte sedimentation rate (77 mm/h). A computed tomographic (CT) scan of the chest showed multifocal bronchiectases in the middle right lobe, multiple small nodules in the lower right lobe, and a cavitary lesion in the upper right lobe, which appeared to have thin walls and was surrounded by a scant or absent inflammatory reaction. Bronchoscopy showed no inflammatory mucosal changes on the right side. Smears of bronchial washings were negative for acid-fast bacilli (AFB), and smears of two sputum specimens were positive, while all specimens gave negative results when tested by a ligase chain reaction commercial assay specific for MTB. A reactivation of tuberculosis was assumed, and chemotherapy with isoniazid, rifampin, and ethambutol was begun. Cultures of bronchial washing and sputum specimens produced a slow-growing *Mycobacterium,* which was identified as *M. celatum* in July 2000. The isolate was not considered clinically important, and chemotherapy was unchanged.

In December 2000, although the general condition of the patient had slightly improved after 6 months of treatment, a control CT scan showed a worsening (enlargement) of the nodular lesions. Smears of two sputum specimens and one bronchial washing specimen were positive for AFB, whereas all specimens were negative when tested by the MTB ligase chain reaction assay. All of these specimens yielded *M. celatum*. On the basis of susceptibility data obtained from the first *M. celatum* isolate, chemotherapy was changed to a schedule that included isoniazid, ethambutol, and clarithromycin, which lasted until November 2001. This regimen proved to be well tolerated by the patient. The bacteriologic results (specimens from two additional bronchial washings were negative for AFB by smear and culture) and clinical response were good. In December 2001, chest x-ray examinations and a CT scan showed a considerable reduction of nodular lesions as well as improvement of lung cavitation (Figure). The patient was considered cured after taking medication for approximately 18 months.

**Figure:**
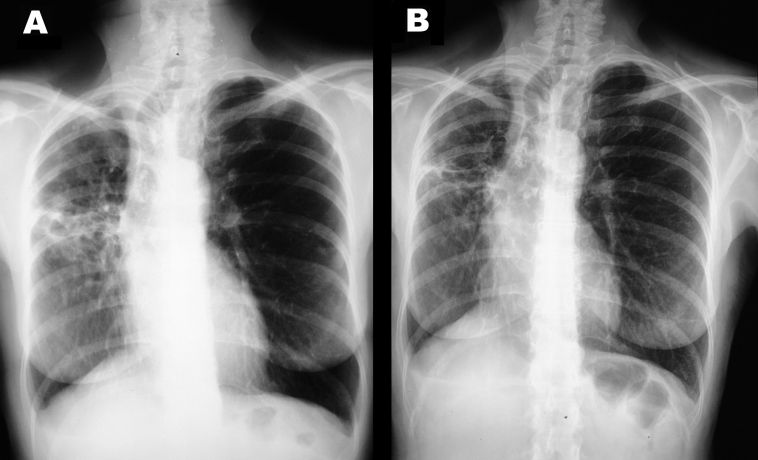
Chest x-ray showing nodular lesions and lung cavitation before (A) and after (B) treatment.

Clinical, microbiologic, and radiologic data as well, as information on response to treatment and follow-up, were obtained from the patient’s records. All material was carefully reviewed to evaluate the clinical significance of the findings according to the criteria proposed by the American Thoracic Society (*14*).

We examined sputum and bronchoaspirate specimens for acid-fast organisms. They were stained with routine Ziehl-Neelsen stain and cultured by standard procedures (*15*) by using a combination of radiometric Bactec system (Becton Dickinson Biosciences, Sparks, MD) and Löwenstein-Jensen medium. Amplification for MTB was performed by the LCx-MTB assay (Abbott, Diagnostics Division, Chicago, IL). Conventional identification and growth tests were performed according to standard methods (*15*). We tested recovered strains with the Accuprobe system using specific probes for MAC and MTB. Assays were run from liquid culture setting the unbound probe hydrolysis incubation time at 5 and 10 min and temperature at 60° ± 1°C (*16*). The amount of chemiluminescence emitted was estimated with a Leader 50 luminometer (Gen-Probe Inc., San Diego, CA, USA) and quantified as relative light units (RLUs). Definitive identification was achieved by mycolic acids high-performance liquid chromatography (HPLC) analysis (*17*). Drug susceptibility pattern was determined in 12B liquid medium (Becton Dickinson Biosciences) by using the radiometric macrodilution method developed for MAC (*18*).

Six isolates were recovered in a 6-month period. *M. celatum* was isolated from different specimens: two isolates were from bronchial washing and four from sputum. Acid-fast smears were positive in five of the six specimens. Cultures on Löwenstein-Jensen medium yielded multiple colonies, ranging from 100–150 CFU to a confluent growth. No additional microorganisms were isolated during the admission and follow-up period. Amplification tests performed on all respiratory specimens were repeatedly negative for MTB on smear samples positive and negative for *M. cel*a*tum.* *M. celatum* strains were isolated on both currently used media, radiometric liquid medium (Bactec 12B) and conventional Löwenstein-Jensen solid medium. We used an extended panel of biochemical and cultural tests for conventional identification. On the basis of these test results, the most likely identification, estimated by a program for the computerized identification of mycobacteria (*19*), appeared to be *M. avium-intracellulare* (Table). All strains isolated from this patient produced a weak yellow pigment in the dark, which grew at 45°C and were negative for arylsulfatase activity when tested after 3 days by the method of Kubica and Rigdon (*20*). The Bactec NAP test (Becton Dickinson Biosciences) did not demonstrate, as expected, any inhibition by para-nitro-α-acetilamino-β-hydroxypropiophenone (NAP). The hybridization test performed with Accuprobe specific for MAC gave negative results. Weak positive or slightly below the cutoff (30,000 RLUs) results were observed with Accuprobe MTB (mean RLU value 51,170; range 28,219–111,365). When selection reagent (unbound probe hydrolysis) incubation time was set at 10 min, *M. celatum* strains clearly showed negative results (range 5,721–9,574; mean RLU value 7,210). A reference strain (*M. tuberculosis* ATCC 25177), run in the hybridization assay as a positive control, gave RLUs of 580,321, about 20 times the cut-off value. Finally, the strains were sent to the Mycobacteria Reference Centre in Florence, Italy (Dr. E. Tortoli), and studied by HPLC analysis of mycolic acids. They showed the same profile that allowed all of our strains to be attributed to *M. celatum*.

**Table:** Biochemical analysis of the described isolate (*Mycobacterium celatum*) compared to those of *M. avium* complex and *M. xenopi*

Characteristics	Results^a^ for:		
	Our isolate	*M. avium* complex	*M. xenopi*
Niacin	**–**	**–**	**–**
Nitrate reduction	**–**	**–**	**–**
Thermostable catalase	+	+	+
Tween 80 hydrolysis (10 days)	**–**	**–**	**–**
Tellurite reduction	+	+	V
Arylsulfatase (3 days)	**–**	**–**	+
Urease	**–**	**–**	**–**
Catalase (over 45 mm of foam)	**–**	**–**	**–**
Photochromogenicity	**–**	**–**	**–**
Scotochromogenicity	+	**–**	+
Growth at 25°C	+	+	**–**
Growth at 37°C	+	+	+
Growth at 45°C	+	**–**	+
MacConkey w/o CV	**–**	**–**	**–**
Tolerance to NaCl (5%)	**–**	**–**	**–**
Tolerance to TCH (5 mg/mL)	+	+	+
Growth rate	Slow	Slow	Slow
Colonial morphologic features	Smooth	Smooth	Smooth

^a^**–** negative; +, positive; v, variable; TCH, thiophene-2-carboxylic acid hydrazide.

MICs (μg/mL) determined for the first isolate were the following: streptomycin, 2.0; isoniazid, 0.5; rifampin, >64.0; ethambutol, 2.5; ciprofloxacin, 1.0; ofloxacin, 1.0; clarithromycin, 1.0; ethionamide, 2.5; and rifabutin, 0.5. Drug susceptibility testing was completed after 6 days.

We searched the English-language literature from 1993 to 2001 for all previously reported cases of pulmonary infections with *M. celatum* in immunocompetent patients. The search yielded two published reports (*12*,*13*). Descriptions of the clinical signs, radiologic findings, and treatment were not always available, however.

Clinical and radiographic features of *M. celatum* pulmonary infection resemble those of tuberculosis and other nontuberculous mycobacterial infections. Cough and weight loss are the main symptoms, while pulmonary infiltrates and extensive cavitations have been described in all published reports. Patients did not suffer from any underlying diseases when pulmonary infection with *M. celatum* was diagnosed and had a strongly positive tuberculin skin test, suggesting immunocompetence. Published data and our findings indicate that *M. celatum* caused pulmonary disease, and not merely colonization, in the affected patients. Indeed, all of these cases met the American Thoracic Society criteria for the diagnosis of nontuberculous mycobacterial disease. The patients displayed radiographic evidence of pulmonary disease that could not be attributed to other causes and that was associated in two of three cases with the repeated isolation of the same mycobacterial strain. By contrast, clinical presentation in immunocompromised patients differs greatly, being characterized by interstitial pulmonary infiltrates or by clinical and micobiologic evidence of disseminated disease (*7*).

*M. celatum* strains were isolated on currently used liquid media in all reported cases (radiometric Bactec 12B and MB/BacT [Organon Teknika B.V., Boxtel, the Netherlands]), while samples from one of the published case-patients did not grow on conventional Löwenstein-Jensen solid medium, and growth has not been reported for samples from the second case-patient. Data from an extended panel of biochemical and cultural tests for conventional identification have not been reported; however, the appearance of a scotochromogenic pale yellow pigment with growth at 45°C and a negative 3-day arylsulfatase test are in full agreement with our present and previous findings (*7*). All strains showed a partial hybridization with Accuprobe MTB, which disappeared after 10 min of incubation with selection reagent. From a practical standpoint, setting the selection time at 10 min is advisable when the Accuprobe identification is performed on a mycobacterial culture grown in a liquid medium. In this situation, the characteristics of mycobacterial colonies cannot be observed and false-positive reactions may lead to misidentification. However, if the Accuprobe assay is carried out on a mycobacterial culture grown on a solid medium, a 5-min selection time is recommended. In this case, the appearance of mycobacterial colonies and their characteristics of growth may help to evaluate Accuprobe results. Thus, a weak positive reaction when testing nontuberculous mycobacteria with the *M. tuberculosis* complex Accuprobe strongly suggests that the organism is likely to be *M. celatum*.

In one case (*13*), a misleading positive reaction occurred when a clinical sample containing *M. celatum* was tested for MTB by the Amplified *M. tuberculosis* direct test (AMTD, Gen-Probe, Inc.). The test amplifies 16S rRNA of *Mycobacterium* species by transcription-mediated amplification (*21*). The resulting amplicons are then detected by a hybridization protection assay by using a probe that targets the same genomic region as the probe of the Accuprobe kit (*22*). In our case, when we tested clinical samples by a different amplification system, no false-positive results potentially leading to a delay of appropriate treatment were reported. Patients (including our case-patient) were given different treatment regimens, with three or four antibiotics, mainly ethambutol, rifabutin, clarithromycin, and ciprofloxacin. Clinical improvement, as defined by resolution of symptoms and improved radiographic findings (infiltrates and cavitary lesions), was obtained within 6 months after initiation of therapy in two of three patients. One patient who received antimycobacterial therapy died 10 weeks after admission from complications apparently related to the *M. celatum* infection (*12*). Data from necropsy were not reported. Both cured patients showed a marked improvement when clarithromycin was added the chemotherapy regimen; the patient who later died felt better for 3 weeks when his chemotherapy was changed to a new schedule that included clarithromycin. Despite evidence of clinical and radiologic improvement, one patient was still sputum-positive 1 year after the initiation of therapy (*13*). In-vitro susceptibility data (when available) seemed to correlate with patients’ clinical improvement as the MICs of tested drugs were below the serum concentrations achievable during therapy.

Our findings show that *M. celatum* is an infrequent cause of potentially treatable pulmonary disease in immunocompetent subjects. Clinical picture and repeated isolation from respiratory specimens enabled us to consider *M. celatum* strains as clinically relevant in all the patients reported. Treatment with a three- or four-drug combination, including clarithromycin and ethambutol, should result in considerable reduction in the illness associated with this disease. Although at present only 16S rDNA sequencing or mycolic acid HPLC analysis can confirm *M. celatum* identification, the most practical way to get a preliminary identification relies on a positive DNA hybridization signal for MTB at 5 min but negative hybridization at 10 min with the Accuprobe test. Finally, although no major immunologic disorder could be detected in these patients, the host’s defense failure associated with *M. celatum* infection suggests a possible “hidden immunodeficiency” rather than a true immunocompetence. Recent data on the immunology of nontuberculous mycobacterial infections (*23*) seem to support this hypothesis.
